# Teoría de la complejidad en la atención del paciente con dolor musculoesquelético

**DOI:** 10.7705/biomedica.6440

**Published:** 2022-12-01

**Authors:** Mauro Andreu, Pablo Policastro, Tatiana Días, Yolanda Pardo

**Affiliations:** 1 Departamento de Salud, Universidad Nacional de La Matanza, San Justo, Buenos Aires, Argentina Universidad Nacional de La Matanza Universidad Nacional de La Matanza Buenos Aires Argentina; 2 Facultad de Ciencias de la Salud, Universidad de las Américas, Quito, Ecuador Universidad de las Américas Universidad de las Américas Quito Ecuador; 3 KINÉ- Kinesiología Deportiva y Funcional, Buenos Aires, Argentina KINÉ- Kinesiología Deportiva y Funcional Buenos Aires Argentina; 4 Unidad de Kinesiología, Hospital Durand, Buenos Aires, Argentina Hospital Durand Buenos Aires Argentina; 5 Consorcio de Investigación Biomédica en Red, Epidemiología y Salud Pública Barcelona, España Consorcio de Investigación Biomédica en Red Barcelona España; 6 Grupo de Investigación en Servicios Sanitarios, Instituto Hospital del Mar de Investigaciones Médicas, Barcelona, España Hospital del Mar de Investigaciones Médicas Barcelona España; 7 Departamento de Psiquiatría y Medicina Legal, Universidad Autónoma de Barcelona, Barcelona, España Universidad Autónoma de Colombia Universidad Autónoma de Barcelona Barcelona Colombia

**Keywords:** análisis de sistemas, dolor musculoesquelético, modelos de fisioterapia, placebos, efecto nocebo, Systems analysis, musculoskeletal pain, physical therapy modalities, placebos, nocebo effect

## Abstract

Los sistemas no lineales no son susceptibles de ser investigados con métodos reduccionistas. En este sentido, la teoría de la complejidad ofrece un enfoque alternativo para cuantificar la importancia de los factores contextuales en el paciente con dolor musculoesquelético.

El resultado del uso positivo (placebo) o negativo (nocebo) de factores contextuales en el entorno terapéutico, podría ser responsable de gran parte de un componente inespecífico en la eficacia del tratamiento, afectando directamente la calidad de los resultados relacionados con la salud del paciente (por ejemplo, dolor, funcionalidad o satisfacción).

En los últimos años, se ha incrementado la comprensión del valor de estos efectos. A pesar del creciente interés, el conocimiento y el reconocimiento de los efectos terapéuticos, continúan siendo limitados y heterogéneos entre los fisioterapeutas, lo cual reduce su valor traslacional en el campo de la fisioterapia.

El propósito de este estudio es presentar el abordaje el paciente con dolor musculoesquelético desde la perspectiva la teoría de la complejidad.

## Abstract (en)

Nonlinear systems are not susceptible to research with a reductionist approach. In this sense, the complexity theory provides an alternative approach to quantify the importance of contextual factors in patients with musculoskeletal pain. The use of positive (placebo) or negative (nocebo) contextual factors in the therapeutic setting could largely account for the non-specific component of treatment efficacy, directly affecting the quality of patients’ health-related outcomes (e.g., pain, disability, or satisfaction). In recent years, there has been a better understanding of the effects of contextual factors. However, the knowledge and awareness of them is limited and heterogeneous among physical therapists, reducing their translational value in the field of physiotherapy.

The purpose of this essay is to describe the management of patients with musculoskeletal pain from the complexity theory perspective.

Las ciencias de la salud están en constante cambio y el dinamismo conceptual y físico de las enfermedades no debe considerarse meramente como un proceso biocientífico, sino que también implica una amplia gama de experiencias humanas y sociales ^1^. En estudios recientes se ha demostrado que la complejidad se origina en la interacción entre los propios factores del paciente y otros factores contextuales ^2^. Sin embargo, las ciencias de la salud, al igual que muchas otras disciplinas científicas, aún optan por métodos clásicos (como el cartesiano) que representan procesos, análisis e interpretaciones desde una perspectiva reduccionista.

La esencia de la susodicha perspectiva radica en que, un problema o el análisis de un objeto, se divide en sus componentes más pequeños, se examina y, luego, la información recopilada se utiliza para sacar conclusiones sobre la naturaleza de una realidad más amplia.

Es fundamental para este enfoque, que el problema se examine por medio de un sistema lineal ^3^^,^^4^. Cuando este es el caso, el enfoque reduccionista es un gran éxito y el profesional de la salud puede, con razón, sentirse seguro al predecir el resultado de una intervención. Sin embargo, las frustraciones aparecen cuando el problema que deseamos examinar no es un sistema lineal simple, sino que, muestra un comportamiento no lineal. Esta problemática de nuestra incapacidad para predecir el resultado en estas situaciones dio nacimiento a la teoría de la complejidad ^3^.

El dolor es una experiencia subjetiva modulada por una variedad de factores cognitivos, emocionales y sensoperceptivos, que surgen del contexto que rodea a la experiencia dolorosa ^5^^,^^6^. El comprender la complejidad del paciente con dolor musculoesquelético es una de las cuestiones más desafiantes, aunque en gran medida poco explorada, pues, los sistemas de salud actuales todavía se centran en las enfermedades individuales y no están equipados para manejar la complejidad ^7^. En los últimos años, se ha incrementado la comprensión del valor de estos efectos terapéuticos. A pesar del creciente interés, su conocimiento y reconocimiento continúan siendo limitados y heterogéneos entre los fisioterapeutas, lo cual reduce su valor traslacional en el campo de la fisioterapia.

El propósito de este estudio es presentar una visión actual de la teoría de la complejidad durante el tratamiento del paciente con dolor musculoesquelético.

## Teoría del caos y sistemas complejos

El desarrollo de la teoría del caos y de la complejidad se dio gracias a la habilidad de las computadoras para llevar a cabo cálculos que rebasan, con mucho, la capacidad del cerebro humano. El experimento clásico de Edward Lorenz formalizó su gestación. Lorenz introdujo variables atmosféricas en un programa computacional, intentando predecir los cambios climáticos. Observó que una minúscula modificación en una de las variables al inicio de los cálculos (en el rango de milésimas de unidad), daba como resultado un algo totalmente diferente ^8^. Esta sensibilidad a las condiciones de partida, conocida popularmente como el «efecto mariposa», es una manifestación de caos. El caos se define como el comportamiento imprevisible de los sistemas complejos gobernados por leyes deterministas ^9^.

Un sistema complejo puede definirse como una red de factores individuales de cuya interacción dinámica emergen nuevas propiedades del propio sistema, y cuyos resultados observables son diferentes a la suma de sus partes individuales ^2^^,^^10^. En otras palabras, es una colección de factores con libertad para actuar de formas que no siempre son totalmente predecibles, y cuyas acciones están interconectadas de modo que las acciones de un factor cambian el contexto para otros factores ^10^. En este sentido, el paciente con dolor musculoesquelético y su interacción con el entorno terapéutico durante la consulta en fisioterapia, también pueden considerarse como un sistema complejo caracterizado por un alto grado de variabilidad biológica, entropía negativa y orden emergente.

En un sistema complejo, los agentes responden a su entorno mediante el uso de un conjunto de reglas internalizadas que impulsan acción. En un sistema bioquímico, las “reglas” son una serie de reacciones químicas. A nivel humano, las reglas pueden expresarse como instintos, construcciones y modelos mentales. “Explorar las experiencias previas, las expectativas y preferencias del paciente”’ es un ejemplo de una regla internalizada que podría impulsar las acciones de un fisioterapeuta (cuadro 1) ^10^.

**Cuadro 1:** Complejidad en un día común en un consultorio de fisioterapia

Una tarde reingresa un paciente consultando por la misma dolencia por la que había consultado el año anterior. El paciente deseaba repetir el mismo tratamiento que habíamos hecho anteriormente pues, según él, la experiencia previa había resultado positiva. Sin embargo, dicho tratamiento no parecía tener sustento científico de efectividad. El paciente, no totalmente de acuerdo, acepta una nueva propuesta de tratamiento recomendada en la literatura. Después de algunas sesiones, los síntomas no disminuían y el paciente solicitó retomar el tratamiento que previamente había resultado efectivo. En las siguientes sesiones, y habiendo retomado el tratamiento anterior, los síntomas del paciente comenzaron a mejorar. Basándonos en este caso, pareciera que el tratamiento que se opone a la evidencia científica disponible ha triunfado. ¿Es este paciente un caso clínico particular? ¿Existen otros factores que hayan podido influir en la evolución del cuadro clínico del individuo? hayan podido influir en la evolución del cuadro clínico del individuo?

Muchas veces, nos vemos desafiados por situaciones complejas durante el abordaje del dolor musculoesquelético de nuestros pacientes. Con el objetivo de resolverlas, seguimos las recomendaciones de la literatura científica. Sin embargo, muchas veces la evolución clínica tiene desenlaces que son difíciles de entender, pues, los síntomas pueden mejorar o empeorar por muchas razones no relacionadas con el tratamiento ^11^^-^^13^.

De acuerdo con Barochiner J., los resultados terapéuticos podrían ser más satisfactorios, tanto para el paciente como para el profesional, si el abordaje terapéutico se realiza comprendiendo las características del sistema complejo ^14^ (cuadro 2). Estas características son las siguientes ^3^^,^^10^^,^^14^^,^^15^:

**Cuadro 2:** Características de un sistema complejo en la atención del paciente con dolor músculoesquelético

Característica	Ejemplo
Gran número	El paciente con dolor musculoesquelético y el terapeuta que lo atiende están conformados por múltiples subelementos (células, moléculas, átomos) y, a la vez, son parte de otros estímulos y sistemas complejos como el entorno del encuentro terapéutico.
Dinamismo	La alianza terapéutica empática o la comunicación verbal y no verbal entre el terapeuta y el paciente pueden condicionar y cambiar el resultado terapéutico.
Penetrancia	En el encuentro terapéutico, las actitudes y los comportamientos positivos hacia el paciente (por ejemplo, con respecto a las afecciones que refiere el paciente, refuerzos positivos) afectan la expectativa del paciente ante el tratamiento y esto condiciona su resultado terapéutico. A su vez, la comunicación del resultado de un tratamiento a otros pacientes puede condicionar la expectativa de estos hacia el resultado terapéutico (por ejemplo, conversación y experiencias de pacientes en la sala de espera o en el gimnasio de rehabilitación).
Falta de	Un mismo tratamiento puede tener diferentes resultados terapéuticos según
linealidad	el estado biopsicosocial del paciente (por ejemplo, ausentismo laboral por su condición) y del terapeuta que lo atiende (por ejemplo, estrés, burnout).
Interacciones	La expectativa previa ante un tratamiento puede modificarse después de una
recursivas	experiencia con el mismo.
Abiertos	El ambiente confortable (poco ruido, música, fragancias, temperatura), la arquitectura adecuada (casilleros privados, equipamiento moderno, espacioso, ventanales), y el diseño ambiental planificado cuidadosamente (decoraciones, adornos y colores), son aspectos que pueden favorecer el resultado terapéutico.
Falta de	Un dolor musculoesquelético que mejoró después del encuentro terapéutico
equilibrio	puede retornar fácilmente a la condición dolorosa ante diferentes y múltiples estímulos (movimiento, estrés laboral, situación familiar, descanso incorrecto).
Historicidad	Las experiencias previas del paciente (por ejemplo, un resultado positivo o negativo de un tratamiento previo) pueden condicionar el resultado terapéutico (cuadro 1).
Información	El paciente con dolor musculoesquelético y el fisioterapeuta actúan con la
local	información inmediata de la condición del paciente (por ejemplo, el movimiento que activó la aparición del dolor), pero, a su vez, se hallan inmersos en la interacción con otros sistemas.

1. *Gran número de elementos.* Estos tienden a mostrar un importante grado de similitud en los diferentes niveles del sistema ^2^, lo que se conoce como fractales ^14^. Los fractales pueden ser geométricos, es decir, una forma divisible en partes, las cuales son una copia a pequeña escala del todo ^3^. En el dolor musculoesquelético, es esencial evaluar la interacción de los individuos y su entorno, lo que incluye infinidad de variables, como ambiente, clima, entorno laboral, familiar y otros.
2. *Dinamismo.* Por su propia naturaleza de actividad constante, los sistemas complejos nunca se encuentran exactamente en el mismo estado. Con el tiempo, pueden cambiar a un estado diferente, o pueden cambiar permanentemente a un estado nuevo y diferente ^16^. Debido a que los agentes dentro de él pueden cambiar, un sistema complejo puede adaptar su comportamiento a lo largo del tiempo ^17^.
3. *Penetrancia.* Las interacciones entre las partes son simultáneas y transversales. La evolución de un sistema influye y es influenciado por la de otro sistema ^8^. Dado que cada agente y cada sistema está anidado dentro de otros sistemas, todos evolucionando juntos e interactuando, no podemos comprender completamente ninguno de los agentes o sistemas sin hacer referencia a los demás ^18^.
4. *Falta de linealidad.* El comportamiento de un sistema complejo comúnmente no es lineal. Los mismos estados pueden tener diferentes respuestas según las características de los elementos del sistema ^10^. Por ejemplo, en la previsión meteorológica, las leyes fundamentales que gobiernan los gases contienen términos no lineales que conducen a lo que los científicos han llamado: “dependencia sensible a las condiciones iniciales’.’ De modo que, una mínima diferencia en las variables iniciales conduce a enormes diferencias en el comportamiento futuro ^15^^,^^19^. Esa idea es mundialmente conocida como «efecto mariposa», ya que el proverbio popular chino «el aleteo de las alas de una mariposa pueden provocar un tsunami al otro lado del mundo», parece reflejar el hecho de que con pequeñas variaciones iniciales podemos conseguir resultados totalmente inesperados.
5. *Interacciones recursivas.* El sistema se retroalimenta ^15^. Esta retroalimentación puede ser positiva o negativa. La respuesta de un sistema complejo puede presentarse como nuevo estímulo al mismo sistema.
6. *Abiertos.* Los sistemas complejos son abiertos e interactúan con su entorno. No tienen límites precisos y se encuentran en permanente intercambio con el medio que los rodea ^2^. No podremos entenderlos sin considerar que se encuentran “anidados” en otros sistemas, y que todos interactúan y evolucionan conjuntamente.
7. *Falta de equilibrio.* Los equilibrios son temporales. Se requiere una gran cantidad de energía para mantener al sistema en un estado de poca entropía.
8. *Historicidad.* Los sistemas complejos tienen una historia dado que evolucionan en el tiempo ^16^. El pasado es importante para entender el estado actual.
9. *Información local.* Los sistemas complejos actúan con información simple y local de su entorno inmediato. El orden, la innovación y el progreso pueden surgir naturalmente de las interacciones dentro de un sistema complejo; no es necesario que se impongan de forma centralizada o desde el exterior ^10^. En la vida cotidiana surgen muchos comportamientos complejos de reglas relativamente simples en cosas como conducir en tráfico o interactuar en reuniones. Mientras nadie dirige nuestras acciones para tales situaciones, todos sabemos cómo adaptarnos y llegar a donde queremos ir ^7^.

## Abordaje del paciente con dolor musculoesquelético desde la complejidad

Los factores determinantes de la evolución clínica son un fenómeno de interés emergente entre profesionales e investigadores de fisioterapia ^6^. El enfoque de la complejidad reconoce al paciente como un conjunto integral, su contexto social, cultural y ambiental, que moldea la respuesta individual a la enfermedad; en esencia, un sistema de salud centrado en el paciente ^20^. Por otro lado, el sistema complejo entiende a la salud y el padecimiento como lo subjetivo y, a la enfermedad, como lo objetivo; existen estados emergentes de interacciones de arriba hacia abajo y de abajo hacia arriba entre el restrictivo contexto ambiental, sociocultural y económico-político y las recursivas interacciones fisiológicas y psicológicas de redes de funciones celulares y de órganos ^16^.

**Figura 1:**
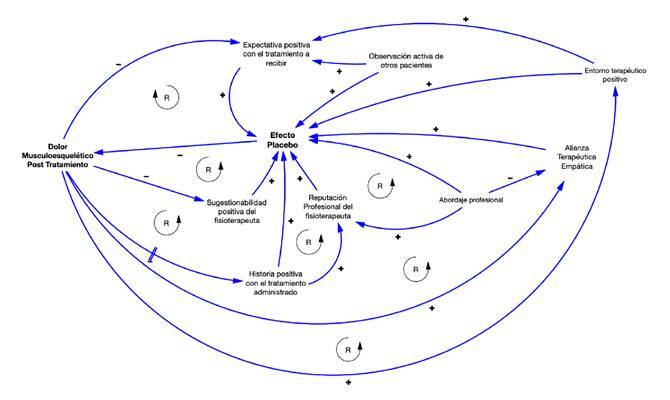
Representación esquemática de algunos factores contextuales que caracterizan la complejidad de la atención del paciente con dolor musculoesquelético. Cada una de las variables mencionadas engloba, a su vez, otras variables interconectadas. Forman, así, una compleja red de sistemas; no es posible alterar una variable sin inﬂuir en todas las demás.

Los factores contextuales son elementos físicos, psicológicos y sociales involucrados en el encuentro clínico entre el paciente y el fisioterapeuta ^21^^,^^22^. En el contexto clínico, la interacción entre el componente específico de un tratamiento y los factores complejos circundantes influencian la experiencia subjetiva terapéutica (por ejemplo, el aumento o la disminución del dolor), desencadenando efectos relacionados con placebo o nocebo ^23^. Específicamente, los factores contextuales positivos pueden mejorar los resultados clínicos, mientras que los factores contextuales negativos pueden amplificar los síntomas del paciente impidiendo su recuperación ^24^. Por ejemplo, un entorno terapéutico positivo puede promover en el paciente una expectativa positiva con el tratamiento que va a recibir, lo que inicia mecanismos que desencadenan efectos placebo que disminuyen el dolor percibido. A su vez, esta disminución del dolor fortalece la alianza terapéutica empática, incrementando el efecto placebo y con mejores resultados terapéuticos (figura 1).

Los efectos placebo y nocebo incorporan fenómenos complejos y distintos en los que se producen cambios conductuales, neurofisiológicos, perceptivos y cognitivos durante el encuentro terapéutico entre el fisioterapeuta y el paciente. Por ejemplo, el ambiente confortable (poco ruido, música, fragancias, temperatura), la arquitectura adecuada (gabinetes privados, equipamiento moderno, espacioso, ventanales) y el diseño ambiental planificado cuidadosamente (decoraciones, adornos y colores), son algunos aspectos que pueden afectar el resultado terapéutico.

Estos efectos también pueden producirse cuando se administra un tratamiento activo y terapéuticamente eficaz. De hecho, cualquier tratamiento (activo o inerte) que se administre en cualquier contexto de atención puede desencadenar efectos relacionados con este contexto. Se pueden identificar factores contextuales relacionados con las características del profesional y del paciente, con la interacción paciente-profesional, con las características del tratamiento y con el entorno de tratamiento ^21^.

### El profesional

La percepción de los pacientes con respecto a la experiencia, el profesionalismo, la apariencia y la reputación del fisioterapeuta tratante, son elementos importantes para el paciente y pueden contribuir a modificar el resultado clínico en las afecciones musculoesqueléticas ^25^.

En un estudio cualitativo se demostró que los pacientes basan sus evaluaciones de calidad de servicio en ciertos aspectos de los fisioterapeutas: sus características personales (comunicación amigable y respetuosa, soporte emocional, sensibilidad a cambios en el estatus del paciente), su disposición a brindar información y educación, y su experiencia técnica ^26^. En una encuesta hecha a los pacientes se informó que el fisioterapeuta vestido con guardapolvo y el fisioterapeuta vestido de camisa, pantalón y corbata fueron clasificados como el más profesional y el más preferido, respectivamente. Por el contrario, los pacientes se mostraron menos satisfechos si vestían jeans. La edad de los encuestados y la exposición con el profesional (número de visitas) afectó la percepción reportada ^27^.

Otros factores, como el incremento en la duración de la sesión y el interés por el paciente y su cuidado, también tienen un rol relevante en la satisfacción del paciente hacia el tratamiento y podrían ser utilizados por los fisioterapeutas para mejorar la satisfacción del paciente ^28^. Los pacientes aprecian la capacidad del fisioterapeuta para animar al paciente a que pregunte, para responder a sus preguntas, para considerar su opinión, para explorar la enfermedad y para conocer la experiencia con la misma; también, su capacidad para brindar una retroalimentación positiva, dar clara información predictiva y explicar sobre la condición del paciente y de cómo el tratamiento puede afectar positivamente los resultados ^28^^-^^31^. Por el contrario, los fisioterapeutas deberían evitar mostrar nerviosismo, dedicar demasiado tiempo a leer las historias clínicas de los pacientes, utilizar demasiadas palabras técnicas o demostrar prisa durante la consulta ^28^^,^^30^.

El carisma del fisioterapeuta y su optimismo o pesimismo con respecto a la naturaleza del tratamiento, pueden tener un efecto en el resultado del tratamiento ^32^. Esta es una “profecía autocumplida” en la cual la expectativa que tiene un profesional sobre el resultado del paciente puede conducir a una mejoría (efecto Pigmalión o Rosenthal) o a un empeoramiento (efecto Golem) del resultado del tratamiento ^33^^,^^34^. Las creencias de los profesionales sobre el dolor de espalda están relacionadas con las creencias de sus pacientes, y las creencias para evitar el miedo en los profesionales se asocian con aquellas para evitar el miedo de sus pacientes ^35^.

### El paciente

La expectativas del paciente son un factor pronóstico importante en el tratamiento del dolor musculoesquelético ^36^ y, frecuentemente, es subestimado por los fisioterapeutas ^37^. Bishop, *et al*., observaron que las expectativas de alivio del dolor tuvieron una influencia importante en el dolor y la incapacidad en pacientes con dolor lumbar ^38^ y dolor cervical ^39^. La experiencia del paciente con un tratamiento previo también ha demostrado ser un factor que puede afectar el resultado de la terapia ^40^.

La percepción de la calidad de la atención fisioterapéutica se experimenta de manera diferente en hombres y mujeres, así como en pacientes de diferentes edades ^41^. Para los pacientes de sexo masculino, los principales factores asociados a la satisfacción con el tratamiento fueron el resultado y las características del fisioterapeuta. Mientras que, en los pacientes de sexo femenino, los principales factores relacionados fueron la organización y la comunicación ^42^^,^^43^.

En otro estudio, la edad del paciente no estuvo asociada con el grado de satisfacción. No obstante, los sujetos mayores de 65 años estuvieron más satisfechos con los factores relacionados al acceso a los servicios de atención y a la efectividad en la comunicación ^44^.

En otro estudio, la satisfacción con la atención fue superior en pacientes con afecciones musculoesqueléticas agudas que en aquellos con afecciones crónicas. Para el grupo con afecciones agudas, las características del fisioterapeuta resultaron las más determinantes de la satisfacción, mientras que, en el grupo con afecciones crónicas, la organización del centro de atención fue el factor predictivo más significativo ^43^.

## La interacción paciente-profesional

Los fisioterapeutas destinan aproximadamente el doble del tiempo en hablar con el paciente que en el tratamiento propiamente dicho ^45^^,^^46^. Factores como escuchar, expresarse positivamente dando apoyo y aliento, el humor y la simpatía, permitir una discusión empática y comunicativa, establecer acuerdos, escuchar la opinión del paciente y utilizar un lenguaje común, son algunas habilidades verbales que se correlacionaron con la satisfacción del paciente y podrían influir significativamente en el resultado del tratamiento ^28^^-^^30^^,^^42^. Por el contrario, los fisioterapeutas deben evitar aspectos como la comunicación negativa, las expresiones verbales de ansiedad y las preguntas cerradas para obtener información ^28^.

Según el estudio hecho por O’Keeffe, *et al*., los pacientes no estuvieron satisfechos con la atención cuando fueron interrumpidos y no pudieron contar su historia, cuando el fisioterapeuta carecía de empatía, cuando era demasiado confiado o cuando se comportaba de manera arrogante ^30^. Los aspectos no verbales, como la expresión verbal y el contacto visual, representan importantes elementos en la alianza terapéutica ^29^.

En el contexto de la atención, los fisioterapeutas intervienen muchas veces con el contacto visual, la sonrisa, y con expresiones de acompañamiento e interés. Además, utilizan regularmente el asentimiento de cabeza, el contacto, la inclinación del cuerpo hacia delante y la orientación corporal para facilitar e involucrar a los pacientes. Todos estos aspectos podrían contribuir al resultado terapéutico final ^45^.

La capacidad del terapeuta para interpretar las expresiones del lenguaje corporal no verbal de los pacientes también es un elemento importante de satisfacción durante el encuentro clínico ^28^.

## El tratamiento

El formular y comentar el diagnóstico de forma detallada, explicar sus afecciones y darle sentido a su afección es *per se* una forma de tratamiento. Estas acciones son apreciadas por los pacientes durante la primera consulta, y podría condicionar el resultado del tratamiento ^29^^,^^42^.

Por otro lado, también es importante adelantar al paciente que el tratamiento que se va a aplicar es importante en la modulación del efecto terapéutico. En un estudio de analgesia posoperatoria, el ocultamiento de la administración de un analgésico resultó en retraso y en menor alivio del dolor, que cuando los pacientes sabían que les estaban administrando el medicamento ^47^. En otro estudio, pacientes con dolor lumbar crónico reportaron menor dolor y más rápida resolución de su condición, cuando realizaron ejercicios de movilización lumbar observándose en un espejo ^48^.

El personalizar el tratamiento, considerar activamente las opiniones de los pacientes y centrar la atención en el paciente parecen influenciar los resultados del tratamiento de manera efectiva y exitosa ^28^^-^^30^^,^^42^. Aspectos como: la atención personalizada y constante con el mismo profesional, la limpieza, el adecuado tiempo de sesión, la puntualidad, la flexibilidad con la agenda personal, la efectividad del tratamiento, la frecuencia y la duración adecuada, afectan positivamente la satisfacción del paciente y el resultado terapéutico ^28^^,^^30^^,^^42^. Por el contrario, el centrar la atención en el profesional, la ausencia de privacidad, los tratamientos costosos, la demora en la entrega de turnos, el escaso tiempo paciente-fisioterapeuta, el ser tratado por diferentes fisioterapeutas, o un tratamiento apresurado, afectan negativamente la satisfacción del paciente y el resultado terapéutico ^28^^,^^30^^,^^42^.

En el contexto clínico, el fisioterapeuta aplica diferentes formas de contacto e interacción al relacionarse con el paciente en los centros terapeúticos, desde la ubicación del paciente en el lugar de la terapia, hasta la socialización de la información y las diversas indicaciones que suceden en la terapia y en la intervención. En el contexto clínico, el fisioterapeuta contacta de diferentes formas al paciente, para asistirlo, para posicionarlo y para informarlo, para percibir información, y para realizar una intervención ^49^. El contacto es un elemento fundamental en las relaciones interpersonales y regula los vínculos sociales ^50^.

## El entorno terapéutico

Diferentes elementos sensoriales del ambiente pueden condicionar el resultado del paciente. La iluminación natural, poco ruido, música suave y relajante, uso de aromas y una temperatura adecuada, son factores importantes que podrían ser considerados en el contexto terapéutico ^51^^,^^52^.

Los aspectos estructurales también parecen ser importantes para el paciente: accesibilidad, horarios convenientes de atención, ubicación, estacionamiento y personal complementario accesible ^42^.

Los ambientes que incluyen muchas ventanas en el lugar de trabajo y los casilleros privados y cómodos son de lo más apreciado por los pacientes ^51^^,^^52^. La decoración de los espacios también podría afectar la percepción del paciente. Encontrando que, las obras de arte sobre la naturaleza, que incluyen vegetación verde, flores, agua y un entorno que integre plantas o adornos de jardín, tienen un efecto relajante ^51^^,^^52^.

## Implicancias

Tradicionalmente, la medicina basada en la evidencia ha focalizado su atención en el efecto de los agentes farmacológicos, subestimando aquellos factores psicológicos y ambientales que contribuyen significativamente a mejorar el resultado terapéutico con nuestros pacientes ^53^. Por tal motivo, es importante considerar el abordaje del paciente con dolor musculoesquelético como un sistema complejo, ya que, ofrecerá oportunidades terapéuticas adicionales para manejar el dolor y podría ser fundamental para mejorar la eficacia terapéutica de diferentes intervenciones.

Es importante reflexionar sobre la importancia de los factores contextuales en el resultado terapéutico, en lugar de minimizarlos o etiquetarlos exclusivamente como factores de confusión ^54^^,^^55^.

Desde una perspectiva educativa, la complejidad todavía es subestimada en la mayoría de las formaciones de grado y posgrado de fisioterapia. No obstante, es un enfoque que, a la luz de la calidad formativa y la profesionalización, así como del conocimiento integro y la bioética, debería incluirse en los programas de estudios. Para garantizar la competencia adecuada, el conocimiento y su uso ético, debería incluirse en los programas de estudios ^21^^,^^56^^-^^58^.

## Conclusión

Los factores determinantes de la evolución clínica son un fenómeno de interés emergente entre profesionales e investigadores del campo de la fisioterapia. El interés en los factores contextuales ha crecido y es ampliamente encontrado en la literatura científica relacionada con la fisioterapia. Para hacer frente a la complejidad en la atención del paciente con dolor musculoesquelético, debemos abandonar los modelos lineales, aceptar la imprevisibilidad, respetar (y utilizar) la autonomía y la creatividad, y responder con flexibilidad a los patrones y oportunidades emergentes.
